# Can anxiety-like behavior and spatial memory predict the extremes of skilled walking performance in mice? An exploratory, preliminary study

**DOI:** 10.3389/fnbeh.2023.1059029

**Published:** 2023-02-28

**Authors:** Aniuska Schiavo, Lucas Athaydes Martins, Luís Eduardo Wearick-Silva, Rodrigo Orso, Léder Leal Xavier, Régis Gemerasca Mestriner

**Affiliations:** ^1^Graduate Program in Biomedical Gerontology, School of Medicine, Pontifical Catholic University of Rio Grande do Sul (PUCRS), Porto Alegre, Brazil; ^2^Neuroplasticity and Rehabilitation Research Group (NEUROPLAR), Pontifical Catholic University of Rio Grande do Sul (PUCRS), Porto Alegre, Brazil; ^3^Developmental Cognitive Neuroscience Lab (DCNL), Pontifical Catholic University of Rio Grande do Sul (PUCRS), Porto Alegre, Brazil; ^4^School of Health and Life Sciences, Pontifical Catholic University of Rio Grande do Sul (PUCRS), Porto Alegre, Brazil

**Keywords:** skilled walking, gait, motor control, anxiety-like, spatial memory, mice

## Abstract

**Introduction:**

Skilled walking is influenced by memory, stress, and anxiety. While this is evident in cases of neurological disorders, memory, and anxiety traits may predict skilled walking performance even in normal functioning. Here, we address whether spatial memory and anxiety-like behavior can predict skilled walking performance in mice.

**Methods:**

A cohort of 60 adult mice underwent a behavioral assessment including general exploration (open field), anxiety-like behavior (elevated plus maze), working and spatial memory (Y-maze and Barnes maze), and skilled walking performance (ladder walking test). Three groups were established based on their skilled walking performance: superior (SP, percentiles ≥75), regular (RP, percentiles 74–26), and inferior (IP, percentiles ≤25) performers.

**Results:**

Animals from the SP and IP groups spent more time in the elevated plus maze closed arms compared to the RP group. With every second spent in the elevated plus maze closed arms, the probability of the animal exhibiting extreme percentiles in the ladder walking test increased by 1.4%. Moreover, animals that spent 219 s (73% of the total time of the test) or more in those arms were 4.67 times more likely to exhibit either higher or lower percentiles of skilled walking performance.

**Discussion:**

We discuss and conclude anxiety traits may influence skilled walking performance in facility-reared mice.

## 1. Introduction

Skilled walking is a highly specialized behavior involving the ability to generate steps, maintain postural balance and adjust movements to accomplish behavioral and contextual/environmental-related demands (Balasubramanian et al., 2014; Geerse et al., 2018). This behavior is controlled by complex sensory-cognitive-motor processes and requires that neural networks overcome the contextual challenges encountered while an individual is moving (Yogev-Seligmann et al., 2008).

The cognitive-motor interplay in humans and animal models is well studied, particularly in stress and neurological disorders (Silva and Frussa-Filho, 2000). For instance, attention and executive functioning deficits are associated with a higher risk of skilled walking impairment (Yogev-Seligmann et al., 2008). Moreover, spatial memory deficits reduce skilled walking performance and increase the likelihood of falls in older adults (Strekalova et al., 2005; Brody and Holtzman, 2006; Kalueff et al., 2007; Anguera et al., 2011) while anxiety can influence skilled movement in people living with Parkinson’s disease (Ehgoetz Martens et al., 2018; Nodehi et al., 2021). Chronic stress can also induce either hyperactivity or hypoactivity in some animals (Heiderstadt et al., 2000). Interestingly, a slight food restriction generated anxiety-like behavior and reduced the movement accuracy in reaching and grasping tasks (Smith and Metz, 2005). Eysenck et al. (2007) found anxiety increased attention regarding perceived threats, but reduced attention when executing a simultaneous task.

Evidence suggests spatial tasks influence postural control, which is the act of maintaining, obtaining, or regaining balance throughout any posture or activity (Pollock et al., 2000). Moreover, previous research in a cross-species model of dual-task walking in young and older humans and rats suggested spatial memory performance is linked with cognitive-motor performance. The findings revealed aged animals and humans performed worse in cognitive-motor tasks that require spatial memory when compared to their younger peers, as observed in dual-task combining object discrimination with the alternation task (Hernandez et al., 2020). Within a broader cognitive-motor interplay, spatial memory contributes to motor control by improving the success of an intended movement in a spatial context (Langan and Seidler, 2011). Moreover, spatial working memory is a good predictor regarding speed of learning when a new skilled motor task is acquired. This is particularly important in the sensorimotor adaptation and in the sequence of a task learning (Anguera et al., 2011).

Although there is a clear link between memory, anxiety disorders and impaired skilled movement, this connection may also be present under physiological conditions. For example, anxiety traits can impact motor performance when healthy people are under pressure to perform an important task/activity (Hutchinson and Cotten, 1973; Wankel, 2014), e.g., when anxiety leads pianists to perform imprecise movements (Kotani and Furuya, 2018). Thus, we hypothesize that extreme percentiles of memory and anxiety-like behavior may be able to predict skilled walking performance. A better understanding of this functional relationship might help predict cognitive-motor disorders, such as Alzheimer (Buchman and Bennett, 2011) or Parkinson-related dementia (Tan et al., 2020). A similar situation could occur among individuals exhibiting borderline memory functioning and anxiety (McDonald, 2011; Gulpers et al., 2016, 2019; Fernandez-Baizan et al., 2019).

To the best of our knowledge, no previous study has addressed whether the spatial memory and anxiety-like behavior can predict skilled walking performance in facility-reared mice. Using the elevated plus maze (to test anxiety-like behavior), Y- and Barnes mazes (to test working and spatial memory), and the ladder walking test (to assess skilled walking performance), we hypothesize that anxiety-like traits might play a role in fine movements, such as those involved in skilled walking. To assess the skilled walking performance, the quality of each paw placement is examined frame-by-frame during the ladder walking test trials and graded using the foot-fault score (Metz and Whishaw, 2002, 2009), thus providing a skilled walking performance metric. Overall, by classifying animals according to their natural skilled walking performance distribution (percentiles ≥75, 74–26, and ≤25), we can determine whether spatial memory or anxiety-like behavior could predict skilled walking performance. Thereby, this research intended to provide essential information for further investigations focused on discovering how neurochemical and genetic factors modulate cognitive-motor interplay in skilled walking.

## 2. Materials and methods

### 2.1. Subjects

We used 60 mice (Balb/cJ, weight, 25–35 g) male (*n* = 28) and female (*n* = 32) acquired from our local colony (CEMBE/PUCRS). The animals were housed in standard lab conditions: same-sex littermates in 3–4 per cage (Tecniplast GM500: 391 mm × 199 mm × 160 mm); water and food *ad libitum*; temperature 23 ± 1°C and 12 h light/dark cycle. This study was approved by the Ethics Committee for Animal Research at the (PUCRS) (number 8955) and was conducted in accordance with the Ethics Guidelines of ICSS and National Institutes of Health Guide for the Care and Use of Laboratory Animals (Russell and Burch, 1959).

### 2.2. Procedure

The experiments were replicated twice, using half the sample each time. Animals were tested during the same period of the day (morning) in an experimental room with controlled temperature (23 ± 1°C) and lighting (500 lux). Before starting the behavioral assessment, the animals underwent 7 days of acclimation with the researchers (a daily 5-min handling session per animal). After, a behavioral test battery was performed from post-natal day (P) P60 to P72. Mice performed the open field (P60), elevated plus maze (P61), Y-maze (P62), and ladder walking (P63) tests. The day after (P64), mice began the first of the three stages of the Barnes maze test, which ended on P72. After each trial, all the apparatus were cleaned using a 70% alcohol solution. On each day, the behavioral battery lasted from 4 to 6 h for the entire sample, depending on the scheduled tests. Mice remained in their habitual housing room and were transferred to the testing room in their home cages approximately 30 min before being assessed (the between-room distance was ∼5 m, located on the same floor). The order in which behavioral tests were conducted was based on the degree of complexity of the tests, from lower to higher in accordance with other studies (Võikar et al., 2004; Lad et al., 2010). At P73, the rodents were euthanized by cervical dislocation. Supplementary Figure 1 illustrates the study design.

### 2.3. Apparatus

The following behavioral tests were used in this study:

#### 2.3.1. Open field (spontaneous locomotor activity)

Animals were placed in the center of a squared Plexiglas box (33 × 33 cm) and allowed to explore the apparatus for 5 min. Video recordings were analyzed using the AnyMaze Software (Stoelting Co., Wood Dale, IL, USA), which divided the field into 16 squares (4 central and 12 peripheral). The total distance traveled, time spent in the central and peripheral zones and number of entrances in each zone were measured (Carola et al., 2002; Kraeuter et al., 2019b; Wearick-Silva et al., 2019).

#### 2.3.2. Elevated plus maze (anxiety-like behavior)

The apparatus, elevated 50 cm above the ground, comprised two open (30 cm × 5 cm) and two closed (30 cm × 5 cm × 15 cm) arms accessed from a central platform (5 cm × 5 cm) (Gomes et al., 2022). Animals were placed individually in the center of the maze facing the open arms and allowed to explore for 5 min (Komada et al., 2008; Kraeuter et al., 2019a; Wearick-Silva et al., 2019). The following outcome measures were analyzed using the AnyMaze Software (Stoelting Co., Wood Dale, IL, USA): the distance traveled; the number of entries in each arm (open and closed arms); the time spent in the open arms, closed arms and in the center of the maze. The avoidance index (AI) was also calculated. AI is defined as a percentage measure of the avoidance of the open arms, calculated as previously described (Trullas and Skolnick, 1993). Briefly, the higher the AI the greater the avoidance behavior.


AI(%)=

$$100-\frac{\left(\%\mathrm{time}\mathrm{in}\mathrm{the}\mathrm{open}\mathrm{arms}+\%\mathrm{entries}\mathrm{in}\mathrm{the}\mathrm{open}\mathrm{arms}\right)}{2}$$

#### 2.3.3. Y-maze (working memory)

The Y-shaped apparatus has three plexiglass arms (35 cm long, 5 cm wide, and 10 cm high, at 120° angle from each other) (Kraeuter et al., 2019c; Viola et al., 2019; Orso et al., 2021). Animals were placed individually in arm B and explored arms A and B for 5 min while arm C (the novel arm) remained closed. After, animals were individually placed in the apparatus (arm B) for 2 min with all arms available for exploration. The phases were separated by a 1-min interval (Morgan et al., 2018). Two raters manually evaluated the number of entries and recorded the exploration time in the arms (A, B, and C) (number, time spent, and percentage). The Y-maze is considered as reliable when the test protocols are followed (Gawel et al., 2019). In addition, the intraclass correlation index showed the results obtained by the two raters were reliable (data not shown). The exploration preference was calculated, as follows:


$$\mathrm{Preference}\,\mathrm{Index}\,\left(s\right)=\frac{\mathrm{time}\,\mathrm{in}\,\mathrm{the}\,\mathrm{novel}\,\mathrm{arm}}{120}$$

Spontaneous alternation was considered to have occurred when a mouse entered each of the 3 arms consecutively, not necessarily observing any particular order (Miedel et al., 2017). The percentage (%) of spontaneous alternation was calculated, as follows:


Spontaneousalternation(%)

$$=\frac{\#\,\mathrm{spontaneous}\,\mathrm{alternations}}{\left(\mathrm{total}\,\mathrm{number}\,\mathrm{of}\,\mathrm{arm}\,\mathrm{entries}-2\right)}\times 100$$

#### 2.3.4. Ladder walking test (skilled walking performance)

The apparatus consisted of two sidewalls made of clear Plexiglas (1 m) and metal rungs to create a horizontal ladder (Metz and Whishaw, 2002). While crossing the ladder, mice were filmed using a GoPro Hero 4 (12 Mp/240 frames-per-second). The first two trials were considered habituation and the third trial was analyzed using the foot fault score system (Metz and Whishaw, 2002; Wearick-Silva et al., 2019) by two independent raters. The inter and intra-rater reliability for the ladder walking test are excellent using both rats and mice models (Martins et al., 2022). The position of the metal rungs across the trials (asymmetrical-only pattern) was modified, except for the first habituation trial. The performance scores were calculated as follows:


Normalizedtotalscore(%)=

$$\frac{\mathrm{Combined}\,\mathrm{limbs}\,\mathrm{performance}\,\mathrm{score}}{M\,e\,a\,n\,o\,f\,t\,h\,e\,c\,o\,m\,b\,i\,n\,e\,d\,l\,i\,m\,b\,s\,p\,e\,r\,f\,o\,r\,m\,a\,n\,c\,e\,s\,c\,o\,r\,e\,f\,r\,o\,m\,t\,h\,e\,c\,o\,h\,o\,r\,t}\times 100$$

A normalized total score (%) lower than 100% means the animal performed worse in the ladder walking test when compared to the cohort mean.

#### 2.3.5. Barnes maze (spatial memory)

The apparatus comprised a circular platform (91 cm diameter), with 20 holes spaced uniformly around the perimeter. The Barnes maze is raised 90 cm above the ground, with a moveable escape box hidden under one hole. Visual cues are used to help the animals locate the escape box. The test is divided into three stages: (a) adaptation; (b) acquisition; and (c) testing. During adaptation, the escape box is kept in the same position and rodents typically adopt three different strategies to find it: random, serial, and spatial. During the 4-day adaptation period, once a day, the mice were placed in the center of the apparatus and given 5 min to find the escape box. On the last day of adaptation, all the animals were allowed to remain in the escape box for 2 min. If they failed to enter the escape box within the 5-min period, they were gently placed there for 2 min. The acquisition stage lasted an additional 4 days. Two trials were carried out daily at 15-min intervals. In each trial, the animals were given 3 min to locate the escape box and allowed to stay there for 1 min. If they failed to enter the escape tunnel within the 3-min period, they were gently placed there for 1 min. Primary errors, total errors, primary latency and total latency were counted in each trial by the ratters executing the test. In terms of reliability the Barnes maze is considered valid when the recommended test protocols are followed (Gawel et al., 2019). In the test stage (probe trial), the escape box was removed and the mice were given 180 s to locate the place where the box had been. The number of pokes in each hole, latency until finding the hole where the box had been and errors were counted (Gawel et al., 2019).

### 2.4. Statistical analysis

To calculate the sample size, we used the previous study by Wearick-Silva et al. (2019). The animals were divided into three groups based on the percentiles of performance in the ladder walking test: (1) superior performer (SP, percentiles ≥75) (*n* = 25; female = 13, male = 12), (2) regular performer (RP, percentiles 74–26) (*n* = 16; female = 9, male = 7), and (3) inferior performer (IP, percentiles ≤25) (*n* = 19; female = 10, male = 9). Between-group comparisons were made using the one-way ANOVA and Tukey’s *post-hoc* test (for parametric data) or Kruskal–Wallis and Mann–Whitney tests (for non-parametric data), according to the data distribution. The potential sex-related differences in the studied sample were also assessed using one-way ANOVA (unadjusted analysis) and by entering “sex” as an independent variable in the regression models (adjusted analysis).

In this study, extreme skilled walking performance (higher or lower performance percentiles in the ladder walking test) was defined by collapsing the SP and IP groups (percentiles ≥75 and percentiles ≤25) (*n* = 44; female = 23, male = 21). The predictive accuracy of the measurements of spatial memory and anxiety-related behavior in determining skilled walking performance was evaluated using the receiver operating characteristic (ROC) curve. Quantitative and qualitative Poisson regressions were used to assess the predictive potential of anxiety-like behavior and spatial memory in determining skilled walking performance. This step-by-step approach allowed us to explore the current research question. Statistical analysis was performed using the Statistical Package for the Social Sciences (SPSS) 20.0 (IBM, NY, USA) and Prism GraphPad 6.0 software (La Jolla, CA, USA). *p* ≤ 0.05 was considered significant.

## 3. Results

The overall mice cohort characterization is shown in Supplementary Table 1. No statistically significant differences were found when we tested for possible sex-related behavioral bias using a between-group comparison (ANOVA) (Supplementary Table 2 and Supplementary Figure 2). Nevertheless, the sex was included as an independent variable in the regression models.

### 3.1. Ladder walking test

**FIGURE 1:**
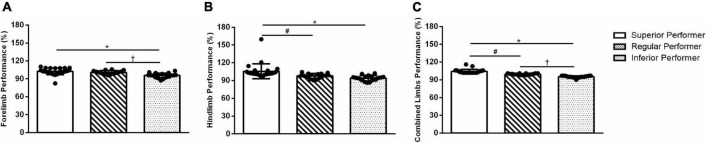
Walking adaptability performance in the ladder walking test. **(A)** Forelimb performance, **(B)** hindlimb performance, and **(C)** combined limbs performance. Data are expressed as mean ± SD. One-way ANOVA and Tukey’s *post-hoc* tests were used. *Superior performers vs. inferior performers; ^#^superior performers vs. regular performers; ^†^regular performers vs. inferior performers.

Between-group differences were observed among SP, RP, and IP groups regarding the “combined limbs performance score” (*F*_(2,59)_ = 67.74; *p* = 0.0001). When the “forelimb performance score” was analyzed (*F*_(2,59)_ = 12.51; *p* = 0.0001), we noticed the SP and IP (*p* = 0.0001) and RP and IP (*p* = 0.005) groups differ. In the “hindlimb performance score” (*F*_(2,59)_ = 10.39; *p* = 0.0001), the SP was different from IP (*p* = 0.0001) and RP (*p* = 0.020). However, the IP group did not differ from the RP (*p* = 0.366) (Figure 1). As expected, these results suggest classifying the mice according to their “combined limbs” skilled walking performance percentiles (≥75, 74–26, ≤25) was statistically significant.

### 3.2. Open field test

No statistically significant between-group differences in the open field test variables were found when classifying mice according to their skilled walking performance (percentiles ≥75, 74–26, ≤25 in the ladder walking test) (Supplementary Figure 3). This suggests skilled walking performance distribution was unrelated with any exploratory behavioral pattern in the open field test.

### 3.3. Elevated-plus-maze

**FIGURE 2:**
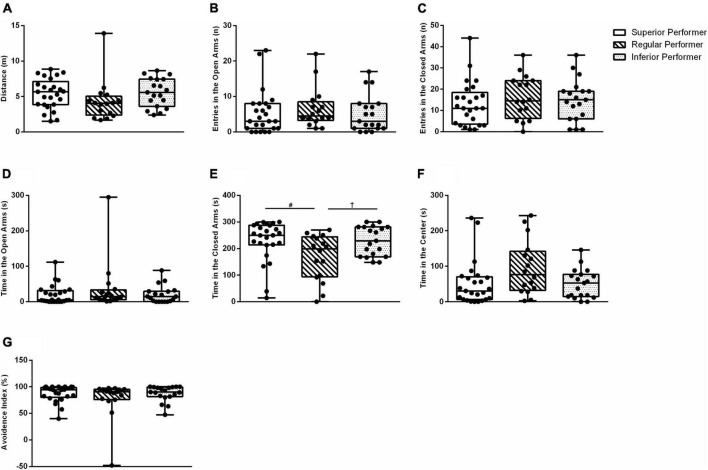
Anxiety-like behavior in the elevated plus maze test. **(A)** Distance traveled, **(B)** number of entries in the open arms, **(C)** number of entries in the closed arms, **(D)** time spent in the open arms, **(E)** time spent in the closed arms, **(F)** time spent in the center, and **(G)** avoidance index. Data are expressed in 25–50–75 percentile and range (minimum and maximum). Kruskal–Wallis and Mann–Whitney tests were used. ^#^Superior performers vs. regular performers; ^†^regular performers vs. inferior performers.

A statistically significant overall difference for “time in the closed arms” was found (KW_(2,59)_ = 7.57; *p* = 0.023) (Figure 2E). The SP and IP groups were different from RP (*p* = 0.008 and *p* = 0.031, respectively). There was no difference between the SP and IP groups (*p* = 0.696) (Figure 2E). All the other assessed outcomes in the elevated plus maze did not differ among the studied groups (Figures 2A–G). In summary, these findings suggest mice exhibiting the higher or lower skilled walking performance percentiles, i.e., extreme skilled walking performance, showed higher levels of anxiety-like behavior in facility-reared mice. Attentional overload can offset some negative effects of anxiety, in which case a task will be adequately performed but at a higher cognitive cost (Smith et al., 2001; Nguyen, 2006). Conversely, anxiety can also make task-irrelevant stimuli more distracting and reduce attentional control, thus resulting in reduced processing efficiency when planning and executing skilled movements (Wilson, 2012). Hence mice exhibiting higher levels of anxiety can exhibit superior or inferior performance in the ladder walking test, which is in line with the literature regarding the attentional control theory. This evidence provide support to collapse SP and IP groups for a preliminary, exploratory analysis.

### 3.4. Y-maze and Barnes maze

**FIGURE 3:**
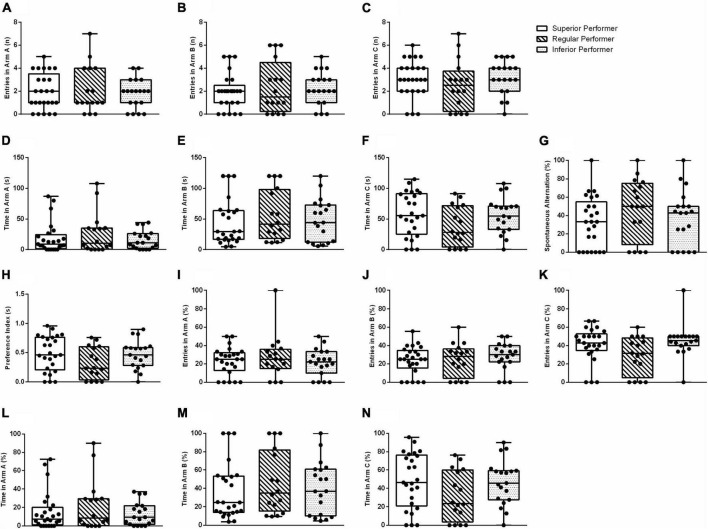
Spatial memory in the Y-maze test. **(A)** Entries in arm A, **(B)** entries in arm B, **(C)** entries in arm C, **(D)** time spent in arm A, **(E)** time spent in arm B, **(F)** time spent in arm C, **(G)** % spontaneous alternation, **(H)** preference index, **(I)** % entries in arm A, **(J)** % entries in arm B, **(K)** % entries in arm C, **(L)** % time spent in arm A, **(M)** % time spent in arm B, and **(N)** % time spent in arm C. Data are expressed in 25–50–75 percentile and range (minimum and maximum). The Kruskal–Wallis and Mann–Whitney tests were used.

No statistically significant between-group differences were found in the Y-maze-assessed outcomes when classifying mice according to their skilled walking performance (percentiles ≥75, 74–26, ≤25 in the ladder walking test) (Figure 3).

The Barnes maze results are shown in Supplementary Figure 4. While we observed a within-group learning effect over time for averaged primary latency (*F*_(4,59)_ = 11.48; *p* = 0.0001), primary error (*F*_(4,59)_ = 7.65; *p* = 0.0001), total latency (*F*_(4,59)_ = 16.93; *p* = 0.0001), as expected, no between-group differences were found when the mice were divided into skilled walking performance percentiles (≥75, 74–26, ≤25 in the ladder walking test). Together, these findings revealed spatial memory did not differ when comparing the mice in terms of skilled walking performance.

### 3.5. Predictive analyses (ROC analysis and Poisson regression)

The accuracy of spatial memory or anxiety-like behavior in predicting regular (RP) or extreme skilled walking performance (SP and IP groups collapsed) were tested using the ROC curve. As mentioned above, the extreme performance category was created because the SP and IP groups did not differ regarding spatial memory or anxiety-like behavior (see group analyses in section “2.4. Statistical analysis”). Moreover, we had an *a priori* suspicion anxiety might play a dual-effect role in skilled walking performance, i.e., higher levels of anxiety-like behavior can trigger different cognitive-motor mechanisms, thus resulting in better or worse skilled movements.

When building the regression models, we tested the outcomes exhibiting *p*-values ≤ 0.20 for group effects. The findings show the elevated plus maze “time in the closed arms” and the Y-maze “entries in arm C (%)” were able to predict the mouse performance in the ladder walking test (*p* < 0.05) (Table 1).

**TABLE 1:** Receiver operating characteristic curve statistics to detect the extreme behaviors in the ladder walking test.

Test/Variable	Area	SD	*p*	95% CI
**(a) Open field**				
Entries in the periphery (%)	0.482	0.079	0.83	0.327–0.638
**(b) Elevated plus maze**				
Time in the closed arms (s)	0.732	0.068	0.01*	0.599–0.864
**(c) Y-maze**				
Entries in arm C (%)	0.692	0.081	0.02*	0.532–0.851
**(d) Barnes maze**				
Search strategy day 4 (mean)	0.434	0.086	0.43	0.265–0.603

SD, standard deviation; *p*, level of significance; 95% CI, 95% confidence interval.

*p ≤ 0.05.

Thereafter, the quantitative Poisson regression model was applied (Table 2). Statistical significance was observed for the variables of the elevated plus maze “time in the closed arms” (*p* = 0.0004) and the Y-maze “entries in arm C (%)” (*p* = 0.002). The quantitative regression findings showed each additional second the mouse spent in the elevated plus maze closed arms increased 1.4% its probability to exhibit an extreme performance in the ladder walking test. Moreover, each additional percentage of entries in Y-maze novel arm increased 4.6% the mouse probability to exhibit an extreme performance in the ladder walking test. The most balanced cut-off points (sensitivity vs. specificity) identified in the ROC curve (Supplementary Tables 3, 4) were used in the qualitative Poisson regression (Table 3). The cut-off point of 219 s (73% of the total time of the test) for the elevated plus maze “time in the closed arms” (sensitivity of 63.6% and specificity of 68.7%) and 42% Y-maze “entries in arm C” (novel arm) (sensitivity 61.4% and specificity 68.7%) were adopted. In the qualitative regression model, the animals that spent 73% of the total time of the test or more in the elevated plus maze closed arms increased by 4.67 times their probability of exhibiting an extreme performance in the ladder walking test. No significant effects were found for the Y-maze “entries in arm C.”

**TABLE 2:** Quantitative regression model to test the relationship between variables of interest with the extreme behaviors in the ladder walking test.

Test/Variable	Exp (B)	*p*	95% CI
**(a) Open field**			
Entries in the periphery (%)	0.898	0.36	0.711–1.133
**(b) Elevated plus maze**			
Time in the closed arms (s)	1.014	0.004*	1.004–1.024
**(c) Y-maze**			
Entries in arm C (%)	1.046	0.02*	1.008–1.086
**(d) Barnes maze**			
Search strategy day 4 (mean)	1.007	0.66	0.975–1.040
**(e) Sex**			
Female (category of reference)	0.882	0.86	0.219–3.561

Exp (B), odds ratio; *p*, level of significance; 95% CI, 95% confidence interval.

*p ≤ 0.05.

**TABLE 3:** Qualitative regression model to test the relationship between the variables of interest and the extreme behaviors of the ladder walking test.

Test/Variable	Exp (B)	*p*	95% CI
**(a) Open field**			
Entries in the periphery (%)	0.899	0.28	0.741–1.091
**(b) Elevated plus maze**			
Time in the closed arms (219 s)	4.672	0.02*	1.226–17.804
**(c) Y-maze**			
Entries in arm C (42%)	2.451	0.19	0.649–9.258
**(d) Barnes maze**			
Search strategy day 4 (mean)	1.007	0.64	0.978–1.036
**(e) Sex**			
Female (category of reference)	0.885	0.86	0.234–3.351

Exp (B), odds ratio; *p*, level of significance; 95% CI, 95% confidence interval.

*p ≤ 0.05.

## 4. Discussion

This exploratory, preliminary study sought to assess whether the anxiety-like behavior and spatial memory could be predictors of skilled walking performance in mice. To the best of our knowledge, this is the first study to explore the issue in a facility-reared mice cohort. Studying a reared-mice cohort is an interesting strategy to explore how spatial memory and anxiety might influence the motor planning needed for controlling skilled walking movements.

Our exploratory, preliminary findings suggest animals exhibiting higher levels of anxiety-like traits are more prone to show extreme skilled walking performance (lower or higher percentiles in the ladder walking test). Thus, the subtle variability in the skilled walking performance in facility-reared mice may be influenced, at least in part, by anxiety-related traits. This is of importance because skilled walking could be impaired by well-known anxiety disorders (Young et al., 2012), but this is the first study to show the anxiety may also influence the control of skilled movements in facility-reared mice.

Increased anxiety levels might produce a dual effect in skilled walking performance due to the anticipatory and/or attentional mechanisms. Some individuals overuse the anxiety-driven cognitive anticipatory mechanism and thus generate a motor plan more likely to fail when facing an irregular walkway/unpredictable obstacle. However, others use anxiogenic inputs to improve their attentional capacity, which results in higher performance levels (Eysenck et al., 2007). The determinants of which individual strategy will be adopted is still unclear.

The attentional control theory states anxiety may cause attentional bias when judging real task-related threats and irrelevant stimuli. As a result, the anxiety-related influences in performance might reflect relative inefficiency in the attentional processes (Eysenck et al., 2007). Higher levels of anxiety may change movement control and compromise movement smoothness (Lohse et al., 2011) as well as impair divided attention tasks in older adults (Hogan, 2003), thus changing the attentional efficiency required to deal with targets in the walkway (Gage et al., 2003). Moreover, there is a strong relationship between stepping inaccuracy and self-reported anxiety. Increased anxiety levels may also influence stepping accuracy indirectly by provoking maladaptive visual sampling strategies (Young et al., 2012). Conversely, some anxiety levels may help improve attention and movement accuracy. One of our most interesting findings is that those mice that stayed 219 s or longer in the elevated plus maze closed arms (longer than the RP group mean) were more likely to exhibit extreme skilled walking performance. This finding is in line with the literature, thus reinforcing that anxiety traits could also influence motor control during skilled walking movements.

Rodents have been used to provide insights into normal and pathological anxiety-like behavior (Van der Staay, 2006). Anxiety is present in both humans and rodents and plays a role in individual defense and survival (Fraser et al., 2010). However, the extent/intensity of anxiety behavior could be influenced by both genetic and phenotype profiles (Smoller and Tsuang, 1998; Meier et al., 2019). Thus, using isogenic mice models (that are genetically identical) may be the best approach to control genetic-based influences. Hence, because we used isogenic mice in this study, we can assume the differences found in anxiety-like behavior are related to the individual’s phenotype. Therefore, the current findings suggest skilled movement is influenced by anxiety-like traits determined by the individual’s phenotype. This also occurs in anxiety disorders, but to a greater extent. Overall, our exploratory, preliminary findings indicate anxiety-like behavior may contribute to determine skilled movement performance. Notwithstanding, fluctuations in anxiety levels may be related to the test conditions or based on intra-individual characteristics (Ramos, 2008). The literature suggests open field, elevated plus maze and light-dark box outcomes may not measure the same type of anxiety-like behavior (Cryan and Holmes, 2005). Similarly, the strain of mice and the apparatus used in the experiments could also influence the measured outcomes (An et al., 2011). For instance, Balb/c mice were found to behave more “anxiously” in the elevated plus maze when compared with the open field test (Trullas and Skolnick, 1993; Carola et al., 2002). Hence, it is not completely unusual to find a lack of concordance between these two tests (Trullas and Skolnick, 1993; Rogers et al., 1999). Moreover, while the open field and elevated plus maze tests are both valuable when assessing anxiety-like behavior, their paradigms are quite different. While open field could be used to assess anxiety-like behavior in general, it is designed to measure exploratory/general locomotion. By contrast, the elevated plus maze is specifically designed to assess anxiety-like behavior (Fraser et al., 2010). Additionally, rearing and grooming were not analyzed in the present study because they could be considered unspecific to identify anxiety levels. While some studies suggest greater rearing counts are associated with anxiety-like behavior in mice, other studies show the opposite (Costall et al., 1989; Borta and Schwarting, 2005). A similar lack of specificity has been reported for grooming counts (Kalueff, 2000; Kalueff and Tuohimaa, 2005).

In this study, the animals were assessed in controlled experimental rooms dedicated to behavioral studies, in the same period of the day, interacting with the same researchers, in accordance with guidelines found in the international literature. Regarding the time spent in different maze zones, the Balb/c mice would naturally be expected to spend more time, on average, avoiding potential risks by remaining more time in the closed arms/peripheral zones of the of the elevated plus maze and open field, respectively (Rodgers and Johnson, 1995; Rodgers and Dalvi, 1997). This may explain the reduced number of crossings from the areas where animals feel safe to areas where they feel more exposed. Different results could have been found if anxiety/stress-induced protocols, anxiolytic treatments, or other mice strains had been used.

The ability to cope with environmental circumstances depends on learning and memory (Vorhees and Williams, 2014). The retention and processing of visuospatial information involves spatial working memory (Fenner et al., 2000). When navigating in a new environment, visuospatial information needs to be temporarily stored and used to locate objects or reach targets, thereby inhibiting distracting stimuli (Flouri et al., 2019). Spatiotemporal parameters of gait are also influenced by working memory (Eysenck et al., 2007) and could be modified during brain aging (Ayoubi et al., 2015). Here, working memory exhibited a subtle capacity to predict skilled walking performance. This may be attributed to the features of the ladder walking test that provide insufficient spatial memory challenges compared with those observed when the rodent is inserted in its ecological context. Although the ladder walking test provides for an asymmetrical pattern, all the rungs have the same diameter, shape and placement level, thus facilitating the animal’s navigation. In addition, the fact the animals underwent three trials on the ladder walking test might have facilitated the creation of an internal image of the test, therefore reducing the demand on the spatial memory (Sorrentino et al., 2019).

This study was designed to minimize biases when establishing behavioral battery assessments, following previously published recommendations (Saré et al., 2021). First, all the experiments were performed in the Center for Experimental Biological Models (Cembe), a reference in animal care and research in southern Brazil. The Center has standard rooms designed and fully dedicated to the study of behavior in mice. Second, all animals undergo a familiarization protocol with the researchers prior to testing; lighting was standardized during the tests and the same researchers conducted all the behavioral tests. Third, a seminal paper from Paylor et al. (2006) using different mice strains demonstrated a 1-day test interval is sufficient to minimize behavioral testing-related effects in comparison with a 1-week test interval, thus supporting the current study design. Finally, several studies have used a similar sequence of behavioral testing (Võikar et al., 2004; Wearick-Silva et al., 2019). Hence, an *a priori* suspicion regarding test interval effects on the current behavioral protocol seems unlikely. Nevertheless, to the best of our knowledge, no previous studies have compared 1-day vs. longer between-test intervals using the same test sequence we adopted.

This study has some limitations. First, skilled walking performance, anxiety, and spatial memory were assessed using different and non-simultaneous tasks. Thus, fluctuations in attention and anxiety-like behavior may have occurred across the tests. Nevertheless, to the best of our knowledge, there is no validated behavioral apparatus capable of simultaneously assessing skilled walking performance, spatial memory, and anxiety-like behavior in rodents. Secondly, we have not compared facility-reared mice with those genetically modified to exhibit anxiety-related disorders. Although such a comparison might be interesting, the link between pathological anxiety and cognitive-motor performance has been well studied (Runswick et al., 2018; Nolte et al., 2019). In addition, brain tissue-related analyses were not possible (due to limited research funding in Brazil), thus the brain mechanisms underlying skilled walking performance were not addressed in this study. Nevertheless, here, our main research goal was to explore whether subtle differences in skilled walking performance could be influenced by anxiety traits. This research contributes to motor control theories, particularly those that sustain personal traits influence the movement strategies adopted to perform some tasks (Bolmont, 2005; Gatto et al., 2011; Berret et al., 2018). Further studies properly designed to compare normal and pathological levels of anxiety-like behavior in mice models using anxiolytic drugs or non-pharmacological strategies may help move the field forward. The influence of anxiety levels on skilled walking performance might differ in physiological and pathological conditions and should be interpreted with caution.

When looking for potential sex-related behavioral bias, we found no changes in the statistical analyses we ran, which is partially consistent with other mouse models (An et al., 2011; Hunsaker et al., 2011). Despite sex (being male or female) having been included as variable in the regression models (see Tables 2, 3), animals were categorized without considering their hormonal status due to the exploratory nature of the study. Nevertheless, we cannot exclude the possibility that sex-related hormones might have influenced the present findings. Although our regression models included “sex” as an independent variable (which was shown not to be significant in the regression models), we did not control the phases of the female estrous cycle. To ensure the acquired data from the female mice would properly represent the behavioral test/test phase under the potential influence of a specific estrous cycle day (proestrus, estrus, metestrus, and diestrus), considering a 13-day long behavioral battery, a very large number of animals would be necessary. Notwithstanding, addressing specific hormonal influences on the different behaviors (independent factors) that may predict walking adaptability performance (dependent factor) could be worthwhile. Hence, our findings could be considered as a starting point to encourage further studies covering this issue.

Additionally, the clustered analysis of males and females, the number of animals used, and the lack of an anxiolytic treatment without motor effects, e.g., allopregnanolone (Hovakimyan et al., 2013; Leppä et al., 2016; Diviccaro et al., 2022) or chrysin (Rodríguez-Landa et al., 2021) could also explain the main results of this preliminary, exploratory study. Hence, further studies properly designed to test the influence of the estrous cycle and anxiolytic-related drugs on anxiety levels and skilled walking performance are required to confirm the current exploratory findings. Several studies have reported that the estrous cycle influences EPM-related behavior (Mora et al., 1996; Galeeva and Tuohimaa, 2001; Marcondes et al., 2001; An et al., 2011; Hunsaker et al., 2011; Scholl et al., 2019). The data analysis in the present study does not measure these cycle phases of the female in the sample, therefore, the current results should be interpreted with caution. In our study, when we assessed a potential between-sex difference for the number of entries in the EPM open arms, a borderline statistical significance was found (*p* = 0.06) (see Supplementary Table 2). When designing trials on anxiety and walking adaptability, verifying within-sex rather than between-sex differences would be advisable (Bale and Epperson, 2017). Our findings, together with those in the literature, suggest the need for further trials addressing sex and hormonal influences on anxiety and skilled walking-related outcomes.

While other behavioral tests could have been adopted in this study, we decided to use only exploratory-based tests without any aversive stimuli. We know from the literature that using aversive stimuli, as seen in Sidman avoidance task or Vogel conflict task, could potentially change mice behavior in other exploratory-based tests (Hiew et al., 2020; Lucantonio et al., 2021). Therefore, when designing a behavioral battery, the minimum number of tests should be used in the same sample (Hånell and Marklund, 2014), as subjecting a sample to an excessive number of tests has been shown to change neurochemical and behavioral findings (Barchas et al., 1978). With this in mind, we considered the widely used elevated plus maze was the most appropriate to assess anxiety-like behavior in the current study (Carola et al., 2002; Yilmazer-Hanke et al., 2003; An et al., 2011; Medeiros et al., 2018; Kraeuter et al., 2019a). Further trials addressing how aversive experiences influence skilled walking performance might benefit from adopting aversive-based tests.

Finally, the lack of any between-group difference in the open field or differences in the time spent in the center or open arms of the elevated plus maze suggest there are subtle anxiety-like behavior changes in facility-reared mice. On the one hand, this is expected because we are not working with models of anxiety disorder but, on the other hand, the lack of consistency in the adopted tests can be considered a study limitation. Further study replication is needed to fully clarify this issue and move the field forward.

## 5. Conclusion

We conclude higher anxiety traits might have a dual effect on skilled walking performance in facility-reared mice, thus predicting, at least in part, the individuals exhibiting superior or inferior performance. This finding agrees with previous research suggesting anxiety traits can modulate cognitive-motor planning when adapting movements such as the ability to adapt walking on an irregular footpath. This exploratory, preliminary study may constitute an important step to encourage further research properly designed to understand the neurobiological mechanisms of skilled walking performance.

## Data availability statement

The raw data supporting the conclusions of this article will be made available by the authors, without undue reservation.

## Ethics statement

This animal study was reviewed and approved by the Ethics Committee for Animal Research at the Pontifical Catholic University of Rio Grande do Sul.

## Author contributions

AS, LM, LW-S, RO, LX, and RM contributed to the conception and design of the study. AS, LM, and RM organized the data and wrote the manuscript. AS and RM performed the statistical analysis. All authors contributed to the article and approved the submitted version.
