# Supplementary material for: Can anxiety-like behavior and spatial memory predict the extremes of skilled walking performance in mice? An exploratory, preliminary study
Source: Front Behav Neurosci. 2023 Feb 28;17:1059029. doi: 10.3389/fnbeh.2023.1059029 (PMC10011164; doi:10.3389/fnbeh.2023.1059029)
Supplement: Supplementary file 1 [file Data_Sheet_1.PDF]

Supplementary Material

1 Supplementary Tables

Supplementary Table 1. Behavioral characterization of the studied mice cohort.

Variables	Mean	Standard Deviation	Median	Minimum	Maximum	Percentiles		
						25	50	75
**Open Field**								
Distance (m)	4.96	3.26	4.23	0.39	15.66	2.08	4.23	7.05
Entries in the Periphery (n)	5.55	7.00	1.50	1.00	28.00	1.00	1.50	8.50
Time in the Periphery (s)	292.99	12.86	298.60	235.80	300.00	290.40	298.60	300.00
Entries in the Center (n)	4.65	7.10	1.00	0.00	28.00	0.00	1.00	7.75
Time in the Center(s)	6.77	12.89	0.40	0.00	64.20	0.00	0.40	9.60
Entries in the Periphery (%)	76.57	23.16	66.67	50.00	100.00	52.28	66.67	100.00
Time in the Periphery (%)	97.66	4.28	99.53	78.60	100.00	96.79	99.53	100.00
Entries in the Center (%)	23.42	23.16	33.33	0.00	50.00	0.00	33.33	47.71
Time in the Center (%)	2.25	4.29	0.13	0.00	21.40	0.00	0.13	3.20
**Elevated Plus Maze**								
Distance (m)	5.16	2.34	5.02	1.52	13.91	3.53	5.02	6.52
Entries in the Open Arms (n)	5.61	5.69	4.00	0.00	23.00	1.00	4.00	8.00
Time in the Open Arms (s)	24.66	42.94	12.50	0.00	294.80	1.85	12.50	29.25
Time in the Center (s)	62.64	63.16	50.40	0.00	243.00	12.92	50.40	86.45
Entries in the Closed Arms (n)	14.53	10.05	14.00	0.00	44.00	6.00	14.00	21.00
Time in the Closed Arms (s)	212.61	77.31	227.75	0.00	300.00	169.02	227.75	273.65
Avoidance Index	84.86	22.38	91.02	-47.90	100.00	80.38	91.02	97.56
**Y-Maze**								
Entries in Arm A (n)	1.95	1.61	2.00	0.00	7.00	1.00	2.00	3.00
Time in Arm A (s)	19.56	24.78	9.95	0.00	108.00	1.88	9.95	30.20
Entries in Arm B (n)	2.16	1.71	2.00	0.00	6.00	1.00	2.00	3.00
Time in Arm B (s)	48.33	36.89	39.05	4.42	120.00	15.58	39.05	70.30
Entries in Arm C (n)	2.98	1.72	3.00	0.00	7.00	2.00	3.00	4.00
Time in Arm C (s)	52.10	33.67	52.77	0.00	115.00	23.01	52.77	75.80
Entries in Arm A (%)	23.61	17.54	25.00	0.00	100.00	14.30	25.00	33.30
Time in Arm A (%)	16.30	20.65	8.30	0.00	90.00	1.55	8.30	25.15
Entries in Arm B (%)	25.36	15.80	27.00	0.00	60.00	17.07	27.00	36.22
Time in Arm B (%)	40.28	30.74	32.55	3.70	100.00	13.02	32.55	58.57

Entries in Arm C (%)	39.36	19.64	42.90	0.00	100.00	32.02	42.90	50.00
Time in Arm C (%)	43.42	28.06	44.00	0.00	95.80	19.15	44.00	63.15
Preference Index (s)	0.43	0.28	0.44	0.00	0.96	0.19	0.44	0.63
Spontaneous Alternation (%)	38.43	29.80	42.86	0.00	100.00	0.00	42.86	60.00
**Ladder Rung Walking Test**								
Forelimb Performance Score (%)	99.96	5.41	100.82	82.54	110.44	96.53	100.82	103.12
Hindlimb Performance Score (%)	99.92	9.99	99.44	86.43	159.69	94.84	99.44	102.16
Combined Limbs Performance Score (%)	100.06	4.58	100.50	90.00	116.00	97.00	100.50	102.00
**Barnes Maze**								
Primary Latency Day 1 (mean)	156.70	40.31	180.00	22.00	180.00	140.87	180.00	180.00
Primary Latency Day 2 (mean)	133.21	56.17	158.25	9.50	180.00	95.62	158.25	180.00
Primary Latency Day 3 (mean)	113.12	60.74	114.50	6.50	180.00	62.00	114.50	180.00
Primary Latency Day 4 (mean)	99.08	58.98	96.50	7.00	180.00	45.00	96.50	154.12
Primary Error Day 1 (mean)	10.32	6.24	9.25	0.00	28.00	5.50	9.25	14.50
Primary Error Day 2 (mean)	9.11	4.66	9.00	0.50	18.50	5.50	9.00	12.87
Primary Error Day 3 (mean)	9.27	6.04	7.50	0.00	26.00	4.62	7.50	14.00
Primary Error Day 4 (mean)	7.18	5.08	5.50	1.00	26.50	3.50	5.50	11.12
Total Latency Day 1 (mean)	178.00	9.30	180.00	121.00	180.00	180.00	180.00	180.00
Total Latency Day 2 (mean)	178.59	17.12	180.00	126.00	280.00	180.00	180.00	180.00
Total Latency Day 3 (mean)	168.23	33.60	180.00	37.00	180.00	180.00	180.00	180.00
Total Latency Day4 (mean)	171.48	114.36	180.00	32.00	990.00	143.25	180.00	180.00
Total Error Day 1 (mean)	11.30	6.31	10.50	2.50	28.00	6.12	10.50	15.75
Total Error Day 2 (mean)	11.12	4.71	10.50	3.00	25.00	8.00	10.50	14.50
Total Error Day 3 (mean)	12.12	6.36	11.50	1.00	28.50	7.62	11.50	16.50
Total Error Day 4 (mean)	9.70	5.48	8.50	1.50	27.00	5.00	8.50	13.50
Search Strategy Day 1 (mean)	0.43	0.44	0.50	0.00	1.50	0.00	0.50	1.00
Search Strategy Day 2 (mean)	0.57	0.46	0.50	0.00	1.50	0.00	0.50	1.00
Search Strategy Day 3 (mean)	0.59	0.54	0.50	0.00	2.00	0.00	0.50	1.00
Search Strategy Day 4 (mean)	0.52	0.39	0.50	0.00	1.00	0.00	0.50	1.00
Total Latency Probe Trial (s)	102.93	118.00	74.21	3.00	180.00	29.00	118.00	180.00
Total Error Probe Trial (n)	6.53	5.00	5.59	0.00	20.00	1.00	5.00	10.75
Search Strategy Probe Trial	0.53	1.00	0.53	0.00	2.00	0.00	1.00	1.00

Supplementary Table 2. Behavioral comparison between male and female mice.

Test / Variables	Female	Male	
**Open Field**	**Median (II 25/75)**	**Median (II 25/75)**	**p**
Distance (m)	4.84 (1.63 / 6.78)	3.80 (2.33 / 7.13)	0.88
Entries in the Periphery (n)	1.00 (1.00 / 7.00)	2.00 (1.00 / 9.00)	0.82
Time in the Periphery (s)	297.50 (290.40 / 300.00)	299.05 (291.10 / 300.00)	1.00
Entries in the Center (n)	0.00 (0.00 / 7.00)	1.00 (0.00 / 8.00)	0.70
Time in the Center (s)	0.00 (0.00 / 9.60)	0.65 (0.00 / 8.83)	0.75
Entries in the Periphery (%)	100.00 (52.29 / 100.00)	66.67 (51.95 / 100.00)	0.61
Time in the Periphery (%)	99.17(96.80 / 100.00)	99.69 (97.03 / 100.00)	1.00
Entries in the Center (%)	0.00 (0.00 / 47.72)	33.33 (0.00 / 48.05)	0.61
Time in the Center (%)	0.00 (0.00 / 3.20)	0.22 (0.00 / 2.95)	0.75
**Elevated Plus Maze**	**Median (II 25/75)**	**Median (II 25/75)**	**p**
Distance (m)	5.19 (3.61 / 6.32)	4.66 (3.18 / 7.53)	0.78
Entries in the Open Arms (n)	2.50 (1.00 / 6.75)	6.00 (3.00 / 8.75)	0.06
Time in the Open Arms (s)	6.25 (1.28 / 26.48)	14.35 (4.28 / 35.05)	0.25
Time in the Center (s)	30.20 (7.43 / 73.75)	59.75 (25.53 / 106.75)	0.09
Entries in the Closed Arms (n)	11.50 (4.25 / 20.50)	16.00 (10.25 / 23.25)	0.10
Time in the Closed Arms (s)	249.70 (192.63 / 281.38)	216.25 (149.50 / 260.13)	0.14
Avoidance Index	93.80 (83.03 / 98.09)	90.33 (76.34 / 95.91)	0.22
**Y-Maze**	**Median (II 25/75)**	**Median (II 25/75)**	**P**
Entries in Arm A (n)	1.50 (0.00 / 3.00)	2.00 (1.00 / 3.75)	0.31
Time in Arm A (s)	9.96 (0.00 / 34.85)	9.85 (2.15 / 26.79)	0.85
Entries in Arm B (n)	2.00 (0.25 / 4.00)	2.00 (1.00 / 3.00)	0.97
Time in Arm B (s)	31.17 (12.75 / 79.57)	43.94 (18.52 / 65.06)	0.81
Entries in Arm C (n)	3.00 (1.25 / 4.00)	3.00 (2.00 / 4.00)	0.52
Time in Arm C (s)	53.68 (17.80 / 84.58)	51.12 (28.24 / 75.33)	0.82
Entries in Arm A (%)	21.10 (0.00 / 32.68)	25.00 (20.00 / 33.30)	0.24
Time in Arm A (%)	8.30 (0.00 / 29.03)	8.20 (1.83 / 22.30)	0.86
Entries in Arm B (%)	29.30 (4.18 / 37.98)	25.00 (18.65 / 33.30)	0.85
Time in Arm B (%)	26.00 (10.60 / 66.30)	36.65 (15.40 / 54.20)	0.81
Entries in Arm C (%)	40.85 (29.15 / 50.00)	44.95 (34.08 / 50.00)	0.36
Time in Arm C (%)	44.75 (14.85 / 70.50)	42.60 (23.55 / 62.75)	0.82
Preference Index (s)	0.44 (0.14/ 0.70)	0.42 (0.23/ 0.62)	0.75
Spontaneous Alternation (%)	41.43 (0.00 / 50.00)	43.65 (20.83 / 64.58)	0.67
**Ladder Rung Walking Test**	**Mean ± SD**	**Mean ± SD**	**P**
Forelimb Performance Score (%)	100.55 ± 4.26	99.30 ± 6.50	0.37

Hindlimb Performance Score (%)	98.72 ± 7.44	101.30 ± 12.29	0.32
Combined Limbs Performance Score (%)	99.81 ± 4.59	100.36 ± 4.64	0.65
**Barnes Maze**	**Median (II 25/75)**	**Median (II 25/75)**	**p**
Primary Latency Day 1 (mean)	180.00 (141.38 / 180.00)	180.00 (137.50 / 180.00)	0.83
Primary Latency Day 2 (mean)	158.25 (126.50 / 180.00)	141.00 (92.63 / 180.00)	0.64
Primary Latency Day 3 (mean)	117.75 (54.13 / 180.00)	110.25 (71.63 / 180.00)	0.97
Primary Latency Day 4 (mean)	96.25 (40.25 / 157.88)	96.50 (45.00 / 146.88)	0.91
Primary Error Day 1 (mean)	9.75 (5.13 / 14.50)	9.00 (5.50 / 14.75)	0.85
Primary Error Day 2 (mean)	10.50 (6.50 / 14.38)	7.75 (4.25 / 9.88)	0.03*
Primary Error Day 3 (mean)	8.00 (4.50 / 14.88)	7.50 (5.13 / 13.38)	0.94
Primary Error Day 4 (mean)	5.00 (2.63 / 9.75)	6.25 (4.50 / 11.88)	0.24
Total Latency Day 1 (mean)	180.00 (180.00 / 180.00)	180.00 (180.00 / 180.00)	0.67
Total Latency Day 2 (mean)	180.00 (180.00 / 180.00)	180.00 (180.00 / 180.00)	0.17
Total Latency Day 3 (mean)	180.00 (180.00 / 180.00)	180.00 (180.00 / 180.00)	0.45
Total Latency Day4 (mean)	180.00 (132.88 / 180.00)	180.00 (163.38 / 180.00)	0.46
Total Error Day 1 (mean)	10.50 (5.63 / 15.63)	10.25 (6.63 / 15.75)	0.82
Total Error Day 2 (mean)	11.25 (9.13 / 15.63)	10.00 (5.63 / 13.50)	0.09
Total Error Day 3 (mean)	11.50 (7.25 / 16.50)	11.25 (7.88 / 17.63)	0.75
Total Error Day 4 (mean)	6.75 (4.00 / 12.75)	12.75 (6.38 / 14.88)	0.01*
Search Strategy Day 1 (mean)	0.50 (0.00 / 1.00)	0.50 (0.00 / 0.88)	0.95
Search Strategy Day 2 (mean)	0.50 (0.00 / 1.00)	0.50 (0.00 / 1.00)	0.75
Search Strategy Day 3 (mean)	0.50 (0.00 / 1.00)	0.75 (0.00 / 1.00)	0.61
Search Strategy Day 4 (mean)	0.50 (0.00 / 1.00)	0.50 (0.13 / 1.00)	0.60
Total Latency Probe Trial (s)	70.50 (15.50 / 180.00)	131.50 (38.00 / 180.00)	0.55
Total Error Probe Trial (n)	4.00 (1.00 / 9.75)	6.50 (3.00 / 11.75)	0.28
Search Strategy Probe Trial	0.50 (0.00 / 1.00)	1.00 (0.00 / 1.00)	0.88

Note: Kruskal Wallis test *p<0,05

Supplementary Table 3. ROC Curve coordinates using the variable 'time in the closed arms' (s) of the Elevated Plus Maze to predict extreme percentiles in the Ladder Walking test.

Cut off Point	Sensitivity	Specificity
171.5500	0.795	0.437
177.0000	0.773	0.437
185.5000	0.750	0.437
194.2500	0.750	0.500
197.7500	0.727	0.500
202.2000	0.705	0.500
208.2500	0.705	0.562
211.5500	0.705	0.625
213.8500	0.682	0.625
216.2500	0.659	0.625
218.1500	0.636	0.625
219.2500	0.636	0.687
220.5000	0.614	0.687
223.7500	0.591	0.687
227.7500	0.568	0.687
233.5000	0.545	0.687
239.5000	0.545	0.750
243.7000	0.523	0.750
247.9500	0.523	0.812
249.7000	0.500	0.812

Supplementary Table 4. ROC Curve coordinates of the variable ‘percentage of entries in arm C’ (new arm) of the Y-Maze to predict extreme percentiles in the Ladder Walking Test.

Cut off Point	Sensitivity	Specificity
32.4500	0.864	0.562
34.5000	0.795	0.562
36.0500	0.773	0.562
36.9500	0.750	0.562
38.7500	0.727	0.562
40.8500	0.636	0.625
42.3000	0.614	0.625
43.3500	0.545	0.687
44.1000	0.545	0.750
44.9500	0.500	0.750
47.7500	0.455	0.750

1.1 Supplementary Figures

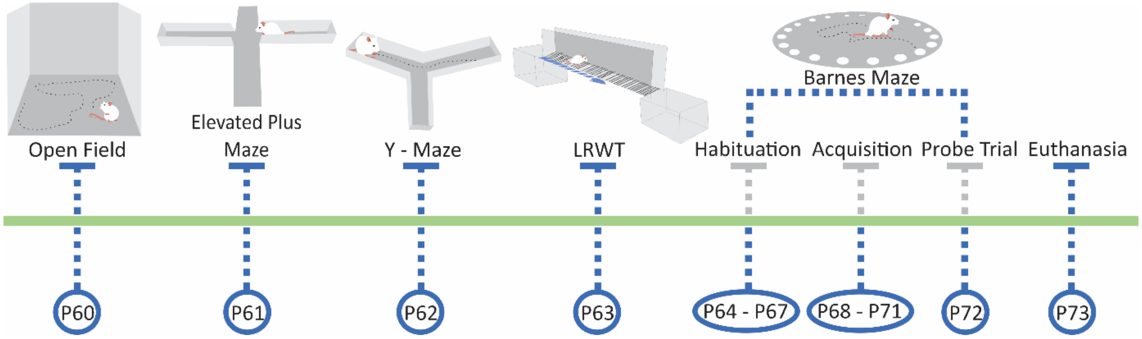

Supplementary Figure 1. Experimental Design.

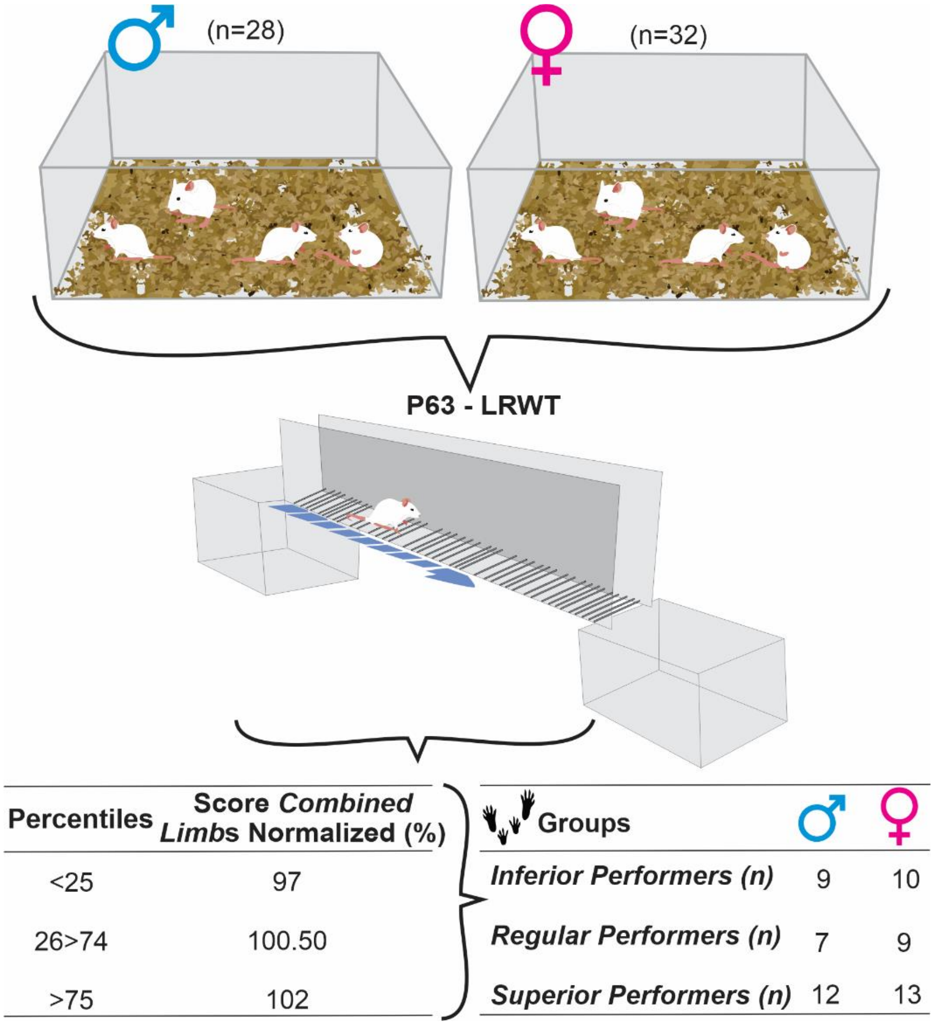

Supplementary Figure 2. Group division (inferior, regular, and superior performers) based on the mice cohort performance (percentiles) in the ladder walking test.

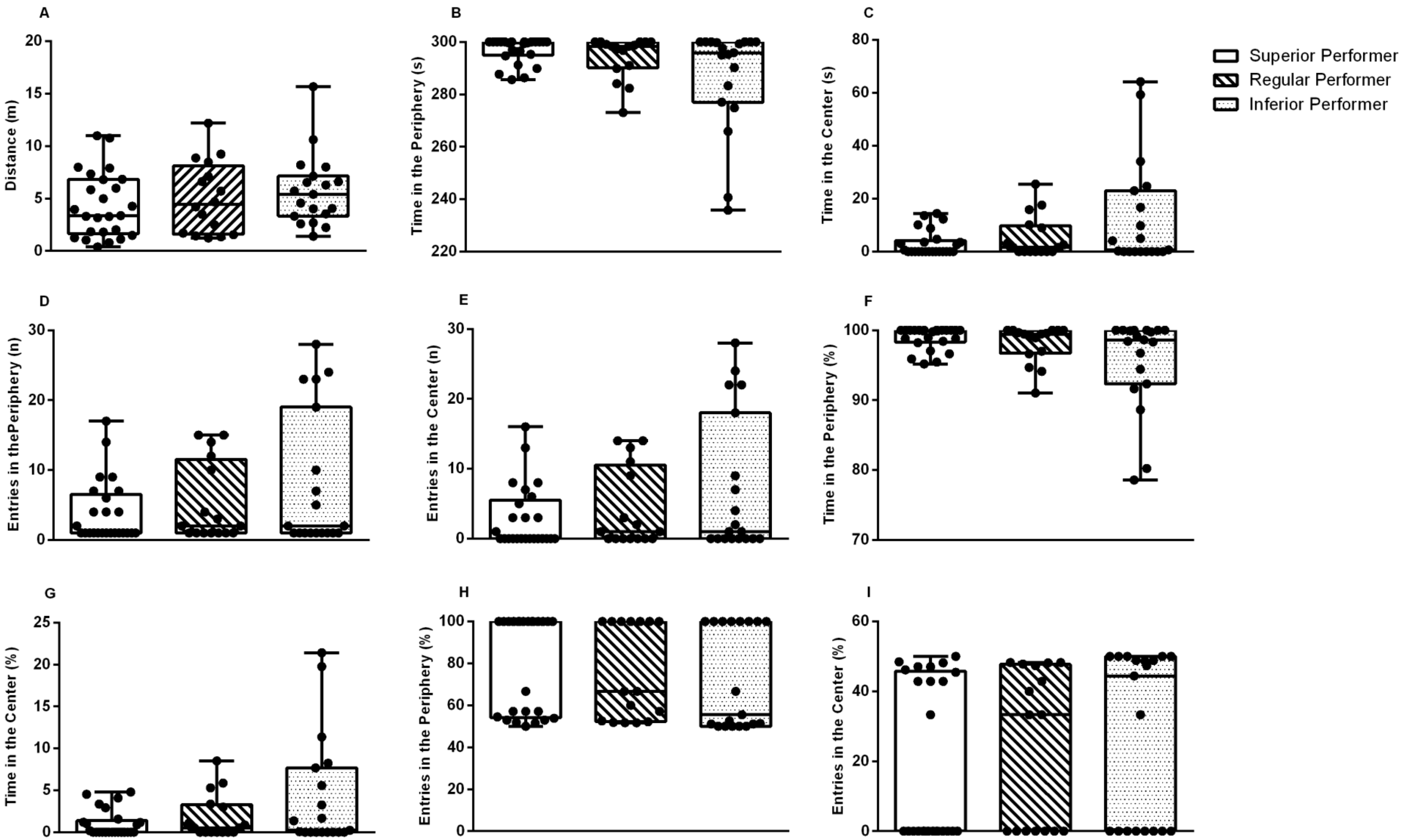

Supplementary Figure 3. Mice locomotion pattern in the Open Field test. (A) Distance traveled, (B) Time spent in peripheral zone, (C) Time spent in the central zone, (D) Number of entries in the peripheral zone, (E) Number of entries in the central zone, (F) % of time spent in the peripheral zone, (G)% of time spent in the central zone, (H) % of entries in the peripheral zone, (I)% of entries in the central zone. Data are expressed in 25-50-75 percentile and range (minimum and maximum). Kruskal Wallis and Mann-Whitney tests were used.

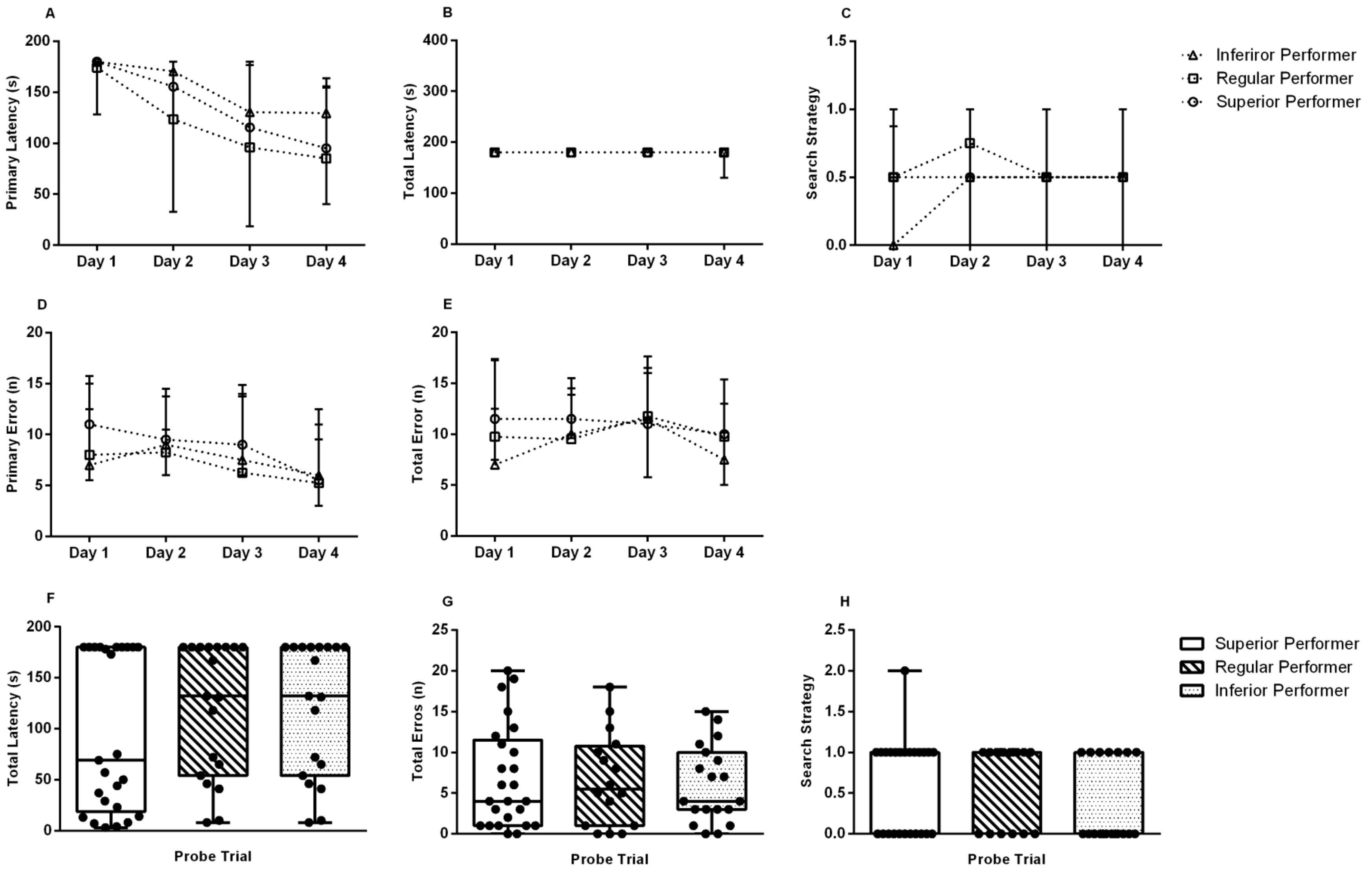

Supplementary Figure 4. Spatial Memory in the Barnes Maze test. (A) Primary latency, (B) Total latency, (C) Search strategy, (D) Number of primary errors, (E) Number of total errors, (F) Probe trial total latency, (G) Probe trial total errors, (H) Probe trial search strategy. Data are expressed in 25-50-75 percentile and range (minimum and maximum). The Kruskal Wallis and Mann-Whitney tests were used.
